# Methane output across life stages in sheep, how it differs from lambs to adult ewes using portable accumulation chambers

**DOI:** 10.1093/jas/skae127

**Published:** 2024-05-08

**Authors:** Edel O' Connor, Nóirín McHugh, Eoin Dunne, Tommy M Boland, Henry Walsh, Norann Galvin, Fiona M McGovern

**Affiliations:** Teagasc, Animal and Grassland Research and Innovation Centre, Athenry, H65 R718, Ireland; School of Agriculture and Food Science, University College Dublin, Belfield, D04 V1W8, Ireland; Teagasc, Animal and Grassland Research and Innovation Centre, Fermoy, P61 P302, Ireland; Teagasc, Animal and Grassland Research and Innovation Centre, Athenry, H65 R718, Ireland; School of Agriculture and Food Science, University College Dublin, Belfield, D04 V1W8, Ireland; Teagasc, Animal and Grassland Research and Innovation Centre, Athenry, H65 R718, Ireland; Teagasc, Animal and Grassland Research and Innovation Centre, Fermoy, P61 P302, Ireland; Teagasc, Animal and Grassland Research and Innovation Centre, Athenry, H65 R718, Ireland

**Keywords:** carbon dioxide, dry matter intake, life stages, portable accumulation chambers, methane, sheep

## Abstract

Methane (CH_4_) produced from enteric fermentation is a potent greenhouse gas produced by ruminant animals. Multiple measurements are required across life stages to develop an understanding of how CH_4_ output changes throughout the animal’s lifetime. The objectives of the current study were to estimate CH_4_ output across life stages in sheep and to investigate the relationship between CH_4_ output and dry matter (DM) intake (DMI). Data were generated on a total of 266 female Suffolk and Texel animals. Methane and carbon dioxide (CO_2_) output, estimated using portable accumulation chambers, and DMI, estimated using the *n*-alkane technique outdoors and using individual penning indoors, were quantified across the animal’s life stage; as lambs (<12 mo), nulliparous hoggets (12 to 24 mo) and ewes (primiparous or greater; > 24 mo). Ewes were further classified as pregnant, lactating, and dry (non-pregnant and non-lactating). Multiple measurements were taken within and across the life stages of the same animals. A linear mixed model was used to determine if CH_4_ and CO_2_ output differed across life stages and using a separate linear mixed model the factors associated with CH_4_ output within each life stage were also investigated. Methane, CO_2_ output, and DMI differed by life stage (*P < *0.05), with lactating ewes producing the greatest amount of CH_4_ (25.99 g CH_4_/d) and CO_2_ (1711.6 g CO_2_/d), while also having the highest DMI (2.18 kg DM/d). Methane output differed by live-weight of the animals across all life stages (*P < *0.001). As ewe body condition score increased CH_4_ output declined (*P < *0.05). Correlations between CH_4_ output measured across life stages ranged from 0.26 (SE 0.08; lambs and lactating ewes) to 0.59 (SE 0.06; hoggets and pregnant ewes), while correlations between CO_2_ output measured across life stages ranged from 0.12 (SE 0.06; lambs and hoggets) to 0.65 (SE 0.06; hoggets and lactating ewes). DMI was moderately correlated with CH_4_ (0.44; SE 0.04) and CO_2_ output (0.59; SE 0.03). Results from this study provide estimates of CH_4_ output across life stages in a pasture-based sheep production system and offer valuable information for the national inventory and the marginal abatement cost curve on the optimum time to target mitigation strategies.

## Teaser

Methane, carbon dioxide output, and dry matter intake differed by life stage with lactating ewes producing the most methane and carbon dioxide while also having the greatest dry matter intake.

## Introduction

Methane (CH_4_) is the second most abundant greenhouse gas in the atmosphere after carbon dioxide (CO_2_); however, CH_4_ has a 27-fold higher global warming potential over a 100-yr period compared to CO_2_ (IPCC, 2021). In the agricultural sector, the dominant source of emissions is CH_4_ produced by the natural process of enteric fermentation (IPCC, 2021). Therefore, the measurement and development of mitigation strategies for CH_4_ output from ruminant animals is becoming increasingly important to help address the climate change challenge (Pickering et al., 2015). Portable accumulation chambers (PAC) have been validated under Irish conditions (O’ Connor et al., 2024) and enable the measurement of large cohorts of animals over a short period in their natural environment (Jonker et al., 2018; O’ Connor et al., 2021a). A profile of an animal’s CH_4_ output at each life stage is required to develop an understanding of how CH_4_ output changes throughout the production cycle from lambs to mature ewes.

A number of factors such as animal stage of production, age, and live-weight (LW) have been shown to effect CH_4_ output in cattle and sheep (Morrison et al., 2017; Oddy et al., 2018); however, whether or not factors affecting CH_4_ output vary across life stages has not yet been investigated in sheep systems. Studies have investigated CH_4_ output across various life stages in cattle (Jiao et al., 2014; Morrison et al., 2017) and sheep (Paganoni et al., 2017; Oddy et al., 2018; Muir et al., 2020) with phenotypic correlations between CH_4_ output across life stages ranging from 0.20 to 0.68 in sheep when measured in PAC and respiration chambers (Oddy et al., 2018; Muir et al., 2020). To date, however, studies have only focused on a select number of life stages (Oddy et al., 2018; Muir et al., 2020), with no study investigating the relationship between CH_4_ output measured in PAC from growing lambs to multiparous ewes. Auxiliary measurements can be taken to coincide with CH_4_ measurements such as dry matter intake (DMI; Oddy et al., 2018) and residual feed intake (RFI; Muir et al., 2020); however, to the best of the authors knowledge, there is no study that has taken auxiliary measurements on DMI, RFI, body condition score (BCS) with information on production traits to coincide with measurement in PAC. The majority of data on DMI from grazing ruminants to coincide with CH_4_ measurements exists for cattle (Wims et al., 2010; O’ Neill et al., 2011) with limited data available on sheep (Ulyatt et al., 2005; Jonker et al., 2017). The objectives of this study, therefore, were to investigate the factors affecting CH_4_ output at each life stage (lambs, hoggets, pregnant, lactating, and dry ewes), to investigate the relationship between CH_4_ output measured across life stages, and to determine the relationship between CH_4_ output and DMI in an Irish sheep production system. Results from this study will not only create a profile of CH_4_ output across pasture-based sheep production cycles from lambs to multiparous ewes but also provides information for national inventory and the marginal abatement cost curve on when mitigation strategies would have the greatest impact.

## Materials and Methods

Data were generated from an experiment carried out over a 3-yr period from 2020 to 2022, inclusive, at the Teagasc Animal and Grassland Research and Innovation Center, Athenry, Co. Galway. The study was approved by the Teagasc Animal Ethics Committee (TAEC0496-2020) and the Health Protection Regulatory Authority (AE19132/P098).

### Animals

A total of 266 purebred females representing two breeds, Suffolk and Texel, were used in this study. Methane, CO_2,_ and DMI measurements were taken both within and across the animals life stage, including as: lambs (<12 mo), nulliparous hoggets (12 to 24 mo), and ewes (primiparous or greater; 24 mo). Ewes were further classified as either: pregnant (11 to 83 d prepartum), lactating (25 to 83 d postpartum), and dry (71 to 565 d postpartum; representing the postweaning period when ewes are non-lactating and non-pregnant). All ewes lambed for the first time at 24 mo of age, with a mean lambing date of mid-March. All animals at each life stage were managed under the same management conditions (described below).

**Figure 1.**
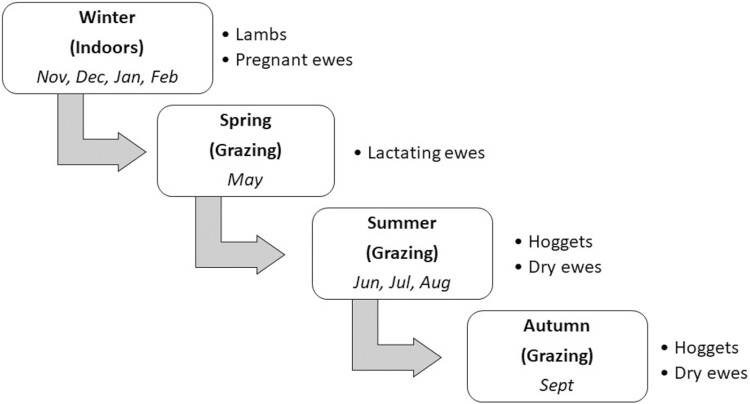
Schematic representation of the seasons and dietary type (outdoor grazing at pasture versus indoor silage-based diet) depicting when measurements in portable accumulation chambers, dry matter intake, live-weight, and body condition score measurements were captured across each life stage (lamb, hogget, pregnant ewe, lactating ewe, and dry ewe).

The Irish lowland sheep production system is predominantly based on a March (spring) lambing flock operating in a pasture-based system. The Irish grazing season commences in spring (February or March) coinciding with lambing and continues until winter (usually November or December) when grass supply diminishes and animals are housed indoors on a grass silage-based diet for approximately 2 to 3 mo. The seasons (winter, spring, summer, and autumn), as well as distinguishing between indoor and outdoor measurements at each life stage are shown in Figure 1. Lamb and pregnant ewe measurements were taken during the winter period when animals were housed indoors and fed a perennial ryegrass silage diet. Nulliparous hogget and lactating and dry ewe measurements were taken outdoors during the grazing season where all animals grazed perennial ryegrass-based swards.

### Methane measurements

PAC were used to obtain CH_4_, oxygen (O_2_), and CO_2_ measurements as described by O’ Connor et al. (2021b). PAC were designed by AgResearch using the Mark III apparatus construction, with 12 individual chambers mounted on a trailer (www.agresearch.co.nz, Steve Gebbie pers. com). The internal volume of each chamber was 853 L (0.77 m long × 1.1 m wide × 1.07 m high) and consisted of an airtight, rectangular-shaped aluminum compartment. A standard protocol was followed to achieve ad libitum access to pasture for all animals, this ensured the grazing height in the paddock was > 5.5 cm on the day prior to measurement in PAC. For measurements that coincided with a grass silage diet, ad libitum access to feed was achieved through the continuous monitoring and placement of feed in front of all animals throughout the day.

On the day of measurement in PAC, all animals were removed from either the paddock or the grass silage diet at 0800 hours and the LW of the animals was immediately recorded using a Prattley weighing scales (O’ Donovan Engineering, Cork, Ireland). All animals were off feed for at least 1 h prior to measurement in PAC (Robinson et al., 2015); with time off feed ranging from 1 to 5 h. Portable accumulation chamber measurements were conducted in lots of 12 animals, with a maximum of six lots measured per day. Animals were randomly assigned to each lot and within each lot of 12 animals, individual animals were randomly assigned to 1 of the 12 individual PAC. Methane (ppm), O_2_ (%), and CO_2_ (%) measurements were obtained over a 50-min measurement period at three specific time points (0, 25, and 50 min) using the RKI Eagle 2 monitor (Weatherall Equipment and Instruments Ltd, UK). Ambient temperature and atmospheric pressure were also recorded in 1-min intervals using the Rotronic Instrument HL-1D Temperature & Humidity Data Logger (Radionics LTD., Dublin, Ireland). Between lots, a gas extraction vacuum system was used to remove all residual CH_4_ and CO_2_. The CH_4_, O_2,_ and CO_2_ measurements obtained from PAC were expressed in grams per day as described by O’ Connor et al. (2021b). A total of 1,726 records on CH_4_ and CO_2_ output were generated for analysis with the number of animals and records per life stage shown in Table 1.

**Table 1.** Number of records and animals (in parenthesis) as well as least square means (± SE) for live-weight (kg), methane (CH_4_) output (g/d), carbon dioxide (CO_2_) output (g/d), dry matter intake (DMI; kg DM/d) and residual feed intake (RFI; kg DM) across each life stage (lambs, hoggets, pregnant ewes, lactating ewes, and dry ewes)

Life stage	Lambs	Hoggets	Pregnant ewes	Lactating ewes	Dry ewes^1^
*PAC measurements*					
No. of records (no. of animals)	238 (120)	303 (152)	118 (59)	351 (151)	716 (246)
Live-weight	55.12 ± 1.61^a^	71.78 ± 1.36^b^	70.47 ± 2.22^b^	77.24 ± 1.34^c^	76.06 ± 0.91^c^
CH_4_ output	11.69 ± 0.99^a^	14.24 ± 0.84^b^	12.28 ± 1.43^ab^	25.99 ± 0.81^c^	21.62 ± 0.53^d^
CO_2_ output	913.54 ± 51.56^a^	977.91 ± 44.13^a^	1144.76 ± 72.92^b^	1711.60 ± 42.38^c^	1345.02 ± 29.21^d^
*Intake measurements*					
No. of records (no. of animals)	58 (58)	118 (59)	59 (59)	111 (111)	97 (96)
DMI	0.87 ± 0.14^a^	1.14 ± 0.10^a^	1.24 ± 0.14^a^	2.18 ± 0.10^b^	1.61 ± 0.08^c^
RFI	−0.009 ± 0.0.57^a^	−0.069 ± 0.306^a^	−0.105 ± 0.525^a^	0.068 ± 0.330^a^	0.067 ± 0.330^a^

^1^Dry ewes represent ewes measured during the postweaning period and are non-lactating and non-pregnant.

^a–d^values within rows with different superscripts differ (*P* < 0.05) from each other.

BCS measurements were taken to coincide with measurements in PAC (±35 d from measurement in PAC) on pregnant, lactating, and dry ewes. BCS was measured on a scale of 1 to 5, in increments of 0.5 (adapted from Russel et al. (1969)), and was measured by the same technician for the duration of the experiment. A total of 1,244 BCS records were available for this study.

### Forage composition

Selective herbage samples, which represented the available herbage for grazing, were collected to coincide with each measurement in PAC at pasture. Representative samples of the silage offered were collected to coincide with indoor measurements in PAC taken during the winter period (Figure 1). To determine dry matter (DM) content both grazing herbage and silage samples were dried at 60 °C for 48 h using a Memmert ‘Excellent’ forced air circulation oven (Memmert GMBH., Schwabach, Germany). Samples were analyzed for ash, neutral detergent fiber (NDF; Van Soest, 1963), acid detergent fiber (ADF; Van Soest, 1963), crude protein (CP; Leco FP‐428; Leco Australia Pty Ltd., Baulkham Hills, New South Wales, Australia) and organic matter digestibility (OMD; Morgan et al., 1989). Results from the analysis of the grazing herbage composition were (mean ± SD): DM%: 19.5% ± 4.1%, Ash: 79.2 ± 12.9 g/kg DM, NDF: 468.2 ± 47.3 g/kg DM, ADF: 229.9 ± 30.4 g/kg DM, CP: 160.9 ± 44.5 kg DM and OMD: 681.1 ± 54.4 g/kg DM. Composition of the silage offered was: DM%: 23.1 ± 5.1% Ash: 88.3 ± 8.0 g/kg DM, NDF: 505.7 ± 53.0 g/kg DM, ADF: 302.3 ± 30.9 g/kg DM and CP: 137.0 ± 30.9 kg DM.

### Dry matter intake

To coincide with CH_4_ measurements (±9 d from CH_4_ measurement), DMI was estimated on a subset of animals across each life stage annually.

#### Outdoor grazing measurements

DMI was estimated on hoggets (summer and autumn), lactating (10 wk postpartum), and dry (18 wk postpartum) ewes, grazing a predominantly perennial ryegrass-based sward using the *n*-alkane technique as described by Mayes et al. (1986) and validated under Irish conditions for grazing sheep by McGovern et al. (2021). Briefly, each animal was administered an *n*-alkane bolus containing 132 mg of C_32_-alkane (*n*-dotriacontane) in the morning for 11 consecutive days. Naturally voided fecal samples were collected from days 7 to 12. Fecal samples were stored at −20 °C until required for further analysis. Fecal samples were then defrosted, bulked per animal, and dried at 40 °C for 48 h or until dry in a Memmert ‘Excellent’ forced air circulation oven. Selective herbage samples were collected from days 6 to 11 to coincide with fecal sample collection. The ratio of herbage C_33_ alkane (tritriacontane) to dosed C_32_ alkane (*n*- dotriacontane) was used to estimate DMI. As fecal samples were bulked per animal, this provided one value of DMI per animal and DMI was expressed as kg DM/d.

#### Indoor housing measurements

DMI was estimated during the winter indoor housing period on lambs and pregnant ewes. For indoor measurements animals were individually penned for a 12-d period and offered a perennial ryegrass silage diet. To ensure ad libitum access to feed, the quantity of daily feed offered to each individual animal was determined based on the animals previous day’s intake plus 10%. Each morning refusals were removed and weighed before animals were fed their daily allocation. DMI was averaged over the measurement period to give one value per animal and DMI was expressed as kg DM/d.

RFI was calculated as the difference between the actual DMI of the animal (based on either the outdoor grazing or indoor housing measurements) and the predicted DMI of the animal. Predicted DMI was estimated using the equation:


$$\begin{array}{l} DMI(kgDMperday)=Date+ADG+MMLW \end{array}$$

where date is the date of *DMI* measurement, *ADG* is average daily gain (calculated over 14 d) and *MMLW* is the metabolic mid-point weight between the start and finishing LW of the animal over the measurement period.

### Auxiliary animal measurements

Across all life stages, data were also available on the age of the animal at time of CH_4_ measurement, animal birth (single, twin, or ≥ triplet) and rearing (single or twin) type, and dam age at rearing in years (2, 3, 4, 5, or ≥ 6). For pregnant ewes scanned litter sizes (single or twin) were also available. Lambing data were also available for lactating and dry ewes which included: number of lambs born (single, twin, or ≥ triplet) and reared (single or twin), ewe age in years (2, 3, or ≥ 4) and the number of days since lambing at the time of CH_4_ measurement.

### Statistical analysis

#### Differences between life stages

To investigate differences in CH_4_ and CO_2_ output, CH_4_ per kg LW (CH_4_ output divided by LW), LW, DMI, and RFI across life stages, a mixed model was undertaken using PROC MIXED (SAS Inst. Inc., Cary, NC) separately for each trait. Life stage (lambs, hoggets, ewes [pregnant, lactating, or dry], and breed [Suffolk and Texel]) were included as fixed effects in all models. When CH_4_ and CO_2_ output and CH_4_ per kg LW were the dependent variables chamber number of measurement, (1 to 12) was included as a fixed effect. Animal was included as a random effect in all models. When the dependent variable was CH_4_ and CO_2_ output or CH_4_ per kg LW the contemporary group was defined as date-lot number of measurement; when the dependent variable was LW, DMI or RFI date represented contemporary group.

#### Factors associated with CH_4_ output within the life stage

A separate series of analyses was performed for each life stage separately to determine factors associated with CH_4_ output within each life stage. Fixed effects considered in all models included: LW, breed, and chamber number of measurement for all life stages, birth (single, twin, or ≥ triplet) and rearing (single or twin) type, and dam age at rearing (2, 3, 4, 5, or ≥ 6) were included as fixed effects for lambs and hoggets. The age of the animal(s; in days) was included as a fixed effect for lambs, hoggets, and pregnant ewes. Pregnancy scanned litter size (single or twin) was included as a fixed effect for pregnant ewes. BCS (2 to 4) was included as a fixed effect for pregnant, lactating, and dry ewes. Birth (single, twin or ≥ triplet) and rearing (single or twin) type of the ewe, ewe age (2, 3, or ≥ 4 yr of age), and number of days since lambing were included as fixed effects for lactating and dry ewes. Contemporary group was included as a random effect in all models.

#### Correlations between gaseous output

To investigate the relationship between CH_4_ and CO_2_ output and CH_4_ and CO_2_ per kg LW measured across life stages and the relationship among CH_4_ and CO_2_ output, O_2_ consumption, LW of the animal and DMI across all life stages, the correlation coefficient was estimated using a generalized linear model in PROC GLM (SAS Inst. Inc.). Fixed effects included breed (Suffolk and Texel), and the chamber number of measurement (1 to 12) and contemporary group included as a random effect.

## Results

### Differences between life stages

In the current study, lambs had the lowest LW at 55.12 ± 1.61 kg (*P < *0.05; Table 1), while the greatest LW associated with lactating (77.24 ± 1.34 kg) and dry ewes (76.06 ± 0.91 kg), which did not differ from each other (*P > *0.05; Table 1). The highest CH_4_ output was associated with lactating ewes (25.99 ± 0.81; *P < *0.05; Table 1), with the percentage difference between CH_4_ output measured in the lactating ewes and all other life stages ranging from 18.36% (dry ewes) to 75.90% (lambs). Similar to CH_4_ output, CO_2_ output was highest in lactating ewes (*P < *0.05; Table 1). There was no statistical difference between CO_2_ output measured in lambs and hoggets, with a percentage difference between CO_2_ output measured in both life stages of 6.81%. Methane per kg LW for each life stage varied from 0.18 g CH_4_/kg LW (pregnant ewes) to 0.34 g CH_4_/kg LW (lactating ewes; *P < *0.05). The highest DMI was associated with lactating ewes (*P < *0.05) followed by dry ewes (*P < *0.05; Table 1). No statistical difference was observed between DMI measured in lambs, hoggets, and pregnant ewes (*P > *0.05; Table 1). RFI ranged from −0.105 ± 0.525 (pregnant ewes) to 0.068 ± 0.330 kg DM (lactating ewes; Table 1) but did not differ across all life stages (*P > *0.05).

### Factors associated with CH_4_ output within the life stage

**Figure 2.**
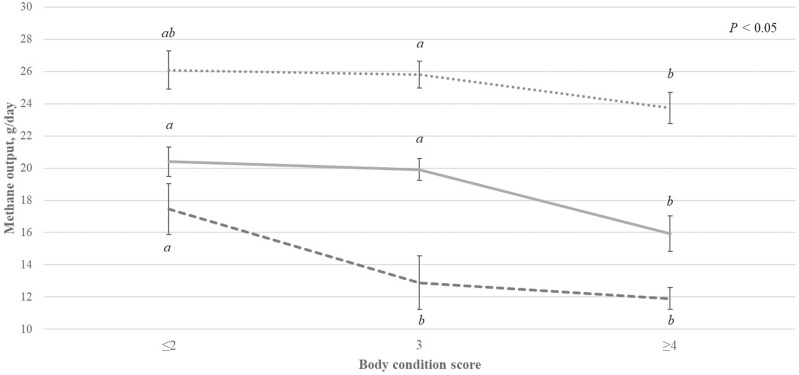
Methane output (g/d; error bars represent one standard error above and below the mean methane output) at different body condition scores for pregnant (broken line), lactating (dotted line), and dry ewes (solid line). ^a-b^ Values within lines with different superscripts differ (*P* < 0.05) from each other.

Factors affecting CH_4_ output at each life stage are shown in Table 2. Methane output across all life stages differed by animal LW (*P < *0.001), with the regression coefficient for LW ranging from 0.13 (dry ewes) to 0.28 (pregnant ewes) g CH_4_/d per unit increase in LW (kg). With the exception of ewes measured during the dry period, chamber number of measurement did not impact CH_4_ output, although differences between chambers were biologically small (on average 2.1 g CH_4_/d). Methane output differed by breed (*P > *0.001) with Texel lambs and pregnant ewes producing, on average, 1.42 g CH_4_/d and 1.85 g CH_4_/day more than their Suffolk counterparts, respectively. Methane output differed by age of the animal (days) in lambs and hoggets (*P < *0.05) but not in pregnant ewes (*P > *0.05; Table 2). For each 1 d increase in age, CH_4_ output increased by 0.04 g/d for lambs (*P < *0.05), but decreased by 0.06 g/d for hoggets (*P < *0.05). In lambs only, CH_4_ output differed by birth and rearing type (*P < *0.001; Table 2), with a percentage difference of 14.30% between singleton-born lambs and twin-born lambs, with a larger percentage difference of 21.77% between singleton-born lambs and triplet born lambs. Lambs that were reared as singletons produced, on average, 2.16 g CH_4_/d less than lambs reared as twins (*P < *0.05). Methane output differed by BCS in pregnant and dry ewes (*P < *0.001) and tended to differ in lactating ewes (*P* = 0.079), with CH_4_ output decreasing as BCS increased (Figure 2). Methane output in lactating ewes differed based on the number of lambs born (*P < *0.05), but not the number of lambs reared (*P > *0.05). Ewes that gave birth to a litter size of three emitted the least CH_4_ output (23.24 g/d), with a percentage difference between ewes that gave birth to a litter size of three and ewes that gave birth to twins or single lambs of 13.89% and 7.38%, respectively. Methane output did not differ by the number of lambs born or reared for ewes measured during the dry period (*P > *0.05; Table 2). Methane output differed by the number of days since lambing in dry ewes only (*P < *0.001) although the differences were biologically small (0.0003 g/d for each day since lambing; *P < *0.01). Methane output differed by the age of the ewe at rearing (years) in dry ewes only (*P < *0.05), with lower CH_4_ output associated with older ewes (Table 2).

**Table 2.** The significance (*P*-value) of the association between factors associated with methane output across and each life stage (lambs, hoggets, pregnant ewes, lactating ewes, and dry ewes)

Factors	Lambs	Hoggets	Pregnant ewes	Lactating ewes	Dry ewes^1^
Chamber number	0.981	0.294	0.562	0.615	0.025
Breed	0.001	0.236	0.003	0.725	0.89
Live-weight, kg	<0.001	<0.001	<0.001	<0.001	<0.001
Age, d	0.026	0.003	0.317	.	.
Age of rearing dam, yr^2^	0.834	0.460	.	0.175	0.003
Birth rank (1, 2 or 3)	0.005	0.545	.	.	.
Rearing rank (1 or 2)	0.001	0.711	.	.	.
Body condition score (≤2, 3, and ≥4)	.	.	0.005	0.079	0.001
Days since lambing	.	.	.	0.248	<0.001
Number of lambs born^3^ (1, 2, or 3)	.	.	0.986	0.030	0.970
Number of lambs reared (1 or 2)	.	.	.	0.604	0.070

^1^Dry ewes represent ewes measured during the postweaning period and are non-lactating and non-pregnant.

^2^Age of the ewe in years was included for lactating and dry ewes.

^3^Number of lambs born includes the scanned litter size for pregnant ewes.

### Within animals correlations between gaseous output across life stages

Weak to moderate correlations were observed between CH_4_ output measured across life stages (Table 3), with correlations ranging from 0.26 (SE 0.06; lambs and lactating ewes) to 0.59 (SE 0.06; hoggets and pregnant ewes). Correlations between CH_4_ output measured across life stages tended to weaken as the gap between life stages increased (Table 3). Correlations between CO_2_ output measured across life stages ranged from −0.03 (SE 0.11; lambs and pregnant ewes) to 0.65 (SE 0.06; hoggets and lactating ewes; Table 3).

**Table 3.** Correlations (SE in parentheses) between methane (CH_4_) output (g/day; above the diagonal), CH_4_ per kg live-weight (LW; below the diagonal) across each animal life stage (lambs, hoggets, pregnant ewes, lactating ewes, and dry ewes)

Life stages	Lambs	Hoggets	Pregnant ewes	Lactating ewes	Dry ewes^1^
Lambs		0.38 (0.05)	0.30 (0.09)	0.26 (0.06)	0.27 (0.06)
Hoggets	0.33 (0.05)		0.59 (0.06)	0.45 (0.07)	0.38 (0.05)
Pregnant ewes	0.39 (0.08)	0.61 (0.06)		0.20 (0.11)	0.39 (0.08)
Lactating ewes	0.18 (0.07)	0.32 (0.08)	0.25 (0.11)		0.32 (0.05)
Dry ewes	0.19 (0.07)	0.35 (0.05)	0.39 (0.08)	0.45 (0.04)	

^1^Dry ewes represent ewes measured during the postweaning period and are non-lactating and non-pregnant.

Correlations between CH_4_ per kg of LW measured across life stages ranged from 0.18 (SE 0.07; lambs and lactating ewes) to 0.61 (SE 0.06; hoggets and pregnant ewes; Table 3). Weak to moderate correlations were observed between CO_2_ per kg of LW measured across life stages and ranged from −0.03 (SE 0.11; lambs and pregnant ewes) to 0.58 (SE 0.04; lactating and dry ewes).

### Correlations among traits

**Figure 3.**
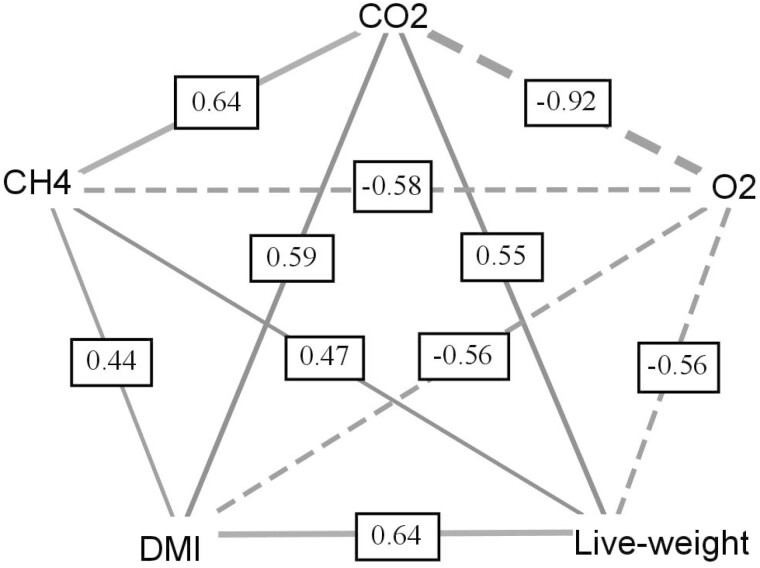
Correlations among methane (CH_4_) output (g/d), carbon dioxide (CO_2_) output (g/d), oxygen (O_2_) consumption (g/d), live-weight of the animal (kg) and dry matter intake (DMI; kg dry matter/d) across all life stages.

The correlations among CH_4_ and CO_2_ output, O_2_ consumption, LW, and DMI across all life stages are shown in Figure 3. Methane output (g CH_4_/d) was strongly correlated with CO_2_ output (0.64; SE 0.01) and negatively correlated with O_2_ consumption (−0.58; SE 0.03). A close to unity correlation (−0.92; SE 0.03) was observed between CO_2_ output and O_2_ consumption. Methane output was moderately correlated with both DMI (0.44; SE 0.04) and LW (0.47; SE 0.02). Similarly, moderate correlations were observed between CO_2_ output and both DMI (0.59; SE 0.03) and LW (0.55; SE 0.02). The correlation between DMI and LW was 0.64 (SE 0.03).

## Discussion

The quantification of CH_4_ output across the animal’s life stages is crucial to develop an insight into changes in CH_4_ output throughout production cycles. Life stage has been proven to impact CH_4_ output in cattle and sheep (Morrison et al., 2017; Oddy et al., 2018), however, whether or not the factors affecting CH_4_ output change across life stages has not yet been investigated. Previous studies have investigated the relationship between CH_4_ output measured across life stages (Paganoni et al., 2018; Muir et al., 2020); however, the same animals were not measured across all life stages from lambs to adult ewes. DMI is commonly measured to coincide with CH_4_ measurements in grazing cattle (Wims et al., 2010; O’ Neill et al., 2011); however, a paucity of studies have measured both traits from grazing sheep (Ulyatt et al., 2005; Jonker et al., 2017). The objectives of this study, therefore, were to investigate the factors associated with CH_4_ output at each life stage, to investigate the relationship between CH_4_ output measured across life stages and to determine the relationship between CH_4_ output and DMI measured in an Irish sheep production system. Results from the present study develop a profile of CH_4_ output across the production cycle of a pasture-based sheep production system.

### Differences between life stages

PAC are predominately used as a ranking tool to identify sheep divergent in CH_4_ rather than to obtain measurements of absolute CH_4_ output (Jonker et al., 2020). Previous studies have reported estimated values for CH_4_ output measured using PAC (Jonker et al., 2018; Muir et al., 2020, 2021), however previous studies have shown that CH_4_ output values measured in PAC are only 31% to 59% of the CH_4_ output measured in respiration chambers (Jonker et al., 2018; O’ Connor, 2024). In the present study, PAC were used to estimate CH_4_ output and showed that CH_4_ output differed across life stages in sheep. DMI is one of the main drivers of CH_4_ output (Swainson et al., 2016); therefore, it was not unexpected that lactating ewes had the highest DMI and CH_4_ output in the present study. Interestingly, only a 5% difference was found between CH_4_ output measured in lambs and pregnant ewes while a larger difference of 35% was found between DMI measured in both these life stages, therefore one would expect that pregnant ewes would have a significantly larger CH_4_ output compared to lambs. However, no statistical difference between CH_4_ output or DMI measured in lambs or pregnant ewes in the current study. Previous studies on pregnant ewes have highlighted that pregnancy is associated with increased digesta flow through the rumen and a reduction in proportion of organic matter lost in the rumen (Weston et al., 1988), therefore, less feed is fermented and less CH_4_ produced from higher intake. This could explain why lower CH_4_ output was observed in the current study for pregnant ewes and is an area that warrants further research.

Methane yield is another parameter that can be used to express CH_4_. Methane yield was not reported in the current study as PAC are a ranking tool for CH_4_ that only gives an estimate of CH_4_ output. Methane yield in the current study ranged from 9.9 to 13.33 g CH_4_/kg DMI and were similar to previously reported values measured using PAC (O’ Connor et al., 2022; O’ Connor, 2024). Values in the current study were lower, however, than values reported using the sulphur hexafluoride technique (15.3 to 22.3 g CH_4_/kg DMI; Pinares-Patiño et al., 2011a; Jonker et al., 2017) and respiration chambers (16 to 24.9 g CH_4_/kg DMI; Pinares-Patiño et al., 2011b; Jonker et al., 2018); which is not expected given that it is comparing a 50 min measurement that is extrapolated up to give a grams per day value to a 24 h measurement period.

### Factors affecting CH_4_ output

Results from previous studies have varied on whether breed of the animal impacts CH_4_ output with differences in breeds reported in cattle and sheep when comparing maternal and terminal breeds (Duthie et al., 2015; Fraser et al., 2015). In the current study CH_4_ output only differed by breed in lambs and pregnant ewes. Two terminal breeds, Suffolk and Texel were used in the present study, with previous studies showing small differences in LW and performance traits between both breeds (Latif and Owen, 1980; McGovern et al., 2020). When LW was removed from the model, the effect of breed on CH_4_ output in pregnant ewes was no longer observed, indicating, that for pregnant ewes, at least, the breed differences could be explained by differences in LW. When DMI was added to the model, the differences in breed were still observed for lambs only, highlighting that differences in CH_4_ output between breeds for lambs, at least, could not be explained by differences in DMI. From this study, differences in CH_4_ output between similar breeds can be observed when animals are in a growing phase of production.

Methane output increased with increasing LW in the current study and is in agreement with studies by Pelchen and Peters (1998) and O’ Connor et al. (2021a) in sheep. This is not unexpected given that heavier animals are associated with higher DMI and therefore, higher CH_4_ output (Flay et al., 2019). Age was found to have a significant effect on CH_4_ output measured in lambs and hoggets in the current study, which corroborates with work by Oddy et al. (2018) that included growing, pregnant, and dry animals. The effect of age is partially due to a reduction in DMI relative to rumen volume with changes in rumen content as the animal gains weight (Oddy et al., 2018) and can explain the effect of age seen in the present study in both lambs and hogget as both life stages are in a growing phase of production.

Methane output decreased as BCS increased, this aligns with previous work conducted on Irish sheep (McGovern et al., 2022). Studies in both cattle and sheep have found negative genetic correlations between BCS and CH_4_ output (Zetouni et al., 2018; Reintke et al., 2020). A linear reduction in DMI is associated with an increase in BCS (Hayirli et al., 2002). When DMI was added to the models in the present study, the differences in BCS were no longer observed, highlighting that differences in BCS could be explained by differences in DMI. This is an area that warrants further investigation. Singleton-born lambs produced more CH_4_ output compared to both twin and triplet-born lambs (*P* < 0.05) while singleton-reared lambs produced 2.16 g/d less CH_4_ compared to lambs reared as twins (*P* < 0.05). When DMI and ADG were added to both of the models, differences between birth and rearing types were no longer observed, highlighting that differences in rearing type could be explained by DMI and ADG of the lambs.

### Within animals correlations between gaseous output across life stages

Methane output was weak to moderately correlated across all life stages in the current study with the correlations declining as the interval between life stages increased. Stronger correlations (0.57 to 0.63) have been reported between growing, pregnant, and dry animals when CH_4_ output was measured in respiration chambers (Oddy et al., 2018) compared to the results reported in the present study. The correlation between CH_4_ output measured in lambs and hoggets in the present study (0.38) was within the range of previously reported values in sheep, 0.20 to 0.40 in PAC (Paganoni et al., 2017; Muir et al., 2020). Weak correlations were reported between lambs and adult ewes in the current study but were similar to previously reported correlations between lambs and adult ewes measured in PAC (0.21 to 0.38; Paganoni et al., 2017; Jonker et al., 2018). While moderate correlations were observed between CH_4_ output measured in adult life stages in the present study. Due to weakening correlations between CH_4_ output measured in lambs and older life stages which differed from zero (*P < *0.05), the significant differences observed between CH_4_ output across life stages and the factors affecting CH_4_ output differing between life stages, for phenotypic analysis if animals are measured at a younger life stage a repeat measurement is required during the adult life stage i.e., once they have reached maturity.

To the best of the authors knowledge, there is a paucity of studies that have investigated the relationship between CO_2_ output measured across life stages using PAC in sheep (Paganoni et al., 2017; Jonker et al., 2018). Carbon dioxide output is a trait of interest as there is the potential to use it as a proxy for DMI and feed efficiency traits (Arthur et al., 2018; Renand et al., 2019). Weak correlations were noted in the present study between CO_2_ output measured in lambs and all other life stages. Carbon dioxide is a byproduct of energy expenditure (Pinares-Patiño et al., 2007). While lambs and hoggets had the lowest CO_2_ output, which did not differ from each other (*P > *0.05), lambs are more likely to move around the chamber throughout the 50-min measurement period compared to the older animals. This may not be a true reflection of the lambs actual CO_2_ output and this may be why the weaker correlations were observed in the current study. Correlations between the other life stages for CO_2_ output were similar to previously reported values which ranged from 0.32 to 0.46 (Paganoni et al., 2017).

### Correlations among traits

DMI is one of the largest sources of variation in CH_4_ output in sheep (Jonker et. al., 2016; Swainson et al., 2016); with previous studies reporting strong correlations between CH_4_ output and DMI when measured in respiration chambers (0.60 to 0.82; Herd et al., 2014; Oddy et al., 2018). A moderate correlation of 0.44 was found between CH_4_ output and DMI in the current study and was within the range of those previously reported when measured in PAC within life stages (0.17 to 0.57; Paganoni et al., 2017; Muir et al., 2020). Measurements in PAC are a point-in-time measurement taken over a 50-min measurement period, where animals have been removed from feed for at least 1 h. The respiration chamber takes multiple measurements across the 24-h measurement period while animals have access to feed throughout the entire measurement period. The discrepancies between both measurement techniques may account for the lower correlation reported between CH_4_ output and DMI measured in PAC compared to the aforementioned respiration chamber studies (Herd et al., 2014; Oddy et al., 2018). Methane output is directly affected by both the LW of the animal and DMI. Therefore, it would be expected that a heavier animal will not only have higher DMI but will also have higher CH_4_ output; which is in agreement with the current study. The correlation between CH_4_ output and LW in the current study was 0.47, which corroborates with previous findings in sheep (Robinson et al., 2010; Moorby et al., 2015; Oddy et al., 2018) and cattle (Donoghue et al., 2015; Ryan et al., 2022).

## Conclusion

Factors associated with CH_4_ output differed across life stages, with LW being the only factor consistently associated with CH_4_ output across all life stages. Results from this study deepen knowledge of the relationship between CH_4_ output and the production traits measured at each life stage. Methane output is weak to moderately correlated across life stages in sheep and for phenotypic analysis, it is recommended to carry out a repeat measurement as an adult ewe when animals are initially measured at younger life stages (i.e., lambs). DMI is positively correlated with CH_4_ output; however, further investigation is required into the relationship between CH_4_ output and DMI at each life stage. Results from this study provide estimates of CH_4_ output across the Irish sheep production cycle from lamb to adult ewe while providing information for the national inventory and the marginal abatement cost curve on the optimum life stage to target mitigation strategies.

## Abbreviations

ADF: acid detergent fiber
ADG: average daily gain
BCS: body condition score
CO_2_: carbon dioxide
CP: crude protein
DM: dry matter
DMI: dry matter intake
LW: live-weight
MMLW: metabolic mid-point weight
CH_4_: methane
NDF: neutral detergent fiber
OMD: organic matter digestibility
O_2_: Oxygen
PAC: portable accumulation chambers
RFI: residual feed intake

## References

[CIT0001] Arthur, P. F., T. Bird-Gardiner, I. M. Barchia, K. A. Donoghue, R. M. Herd. 2018. Relationships among carbon dioxide, feed intake, and feed efficiency traits in ad libitum fed beef cattle. J. Anim. Sci. 96:4859–4867. doi: 10.1093/jas/sky308 30060045 PMC6247828

[CIT0002] Donoghue, K. A., T. L. Bird-Gardiner, P. F. Arthur, R. M. Herd, R. F. Hegarty. 2015. Genetic parameters for methane production and relationships with production traits in Australian beef cattle. In: MacLeod, I., ed. Proceedings of the Association for the Advancement of Animal Breeding and Genetics, September 28–30, 2015. Lorne, VIC, Australia: Association for the Advancement of Animal Breeding and Genetics; p. 114–117.

[CIT0003] Duthie, C. A., J. A. Rooke, J.J. Hyslop, A. Waterhouse. 2015. Methane emissions from two breeds of beef cows offered diets containing barley straw with either grass silage or brewers’ grains. Animal. 9:1680–1687. doi: 10.1017/S1751731115001251 26145179

[CIT0004] Flay, H. E., B. Kuhn-Sherlock, K. A. Macdonald, M. Camara, N. Lopez-Villalobos, D. J. Donaghy, J. R. Roche. 2019. Hot topic: selecting cattle for low residual feed intake did not affect daily methane production but increased methane yield. J. Dairy Sci. 102:2708–2713. doi: 10.3168/jds.2018-15234 30639015

[CIT0005] Fraser, M. D., H. R. Fleming, V. J. Theobald, and J. M. Moorby. 2015. Effect of breed and pasture type on methane emissions from weaned lambs offered fresh forage. J. Agric. Sci. 153:1128–1134. doi: 10.1017/S0021859615000544 26236042 PMC4501301

[CIT0006] Hayirli, A., R.R. Grummer, E. V. Nordheim, P. M. Crump. 2002. Animal and dietary factors affecting feed intake during the prefresh transition period in Holsteins. J. Dairy Sci. 85:3430–3443. doi: 10.3168/jds.S0022-0302(02)74431-7 12512616

[CIT0007] Herd, R. M., P.F. Arthur, K. A. Donoghue, S. H. Bird, T. Bird-Gardiner, R. S. Hegarty. 2014. Measures of methane production and their phenotypic relationships with dry matter intake, growth, and body composition traits in beef cattle. J. Anim. Sci. 92:5267–5274. doi: 10.2527/jas.2014-8273 25349368

[CIT0008] Jiao, H., T. Yan, D. A. Wills, A. F. Carson, D. A. McDowell. 2014. Development of prediction models for quantification of total methane emission from enteric fermentation of young Holstein cattle at various ages. Agric. Ecosyst. Environ. 183:160–166. doi: 10.1016/j.agee.2013.11.004

[CIT0009] Jonker, A., G. Molano, J. Koolaard, S. Muetzel. 2016. Methane emissions from lactating and non-lactating dairy cows and growing cattle fed fresh pasture. Anim. Prod. Sci. 57:643–648. doi: 10.1071/AN15656

[CIT0010] Jonker, A., S. Hickey, C. Pinares-Patiño, J. McEwan, S. Olinga, A. Díaz, G. Molano, S. MacLean, E. Sandoval, R. Harland, D. Birch. 2017. Sheep from low-methane-yield selection lines created on alfalfa pellets also have lower methane yield under pastoral farming conditions. J. Anim. Sci. 95:3905–3913. doi: 10.2527/jas.2017.1709 28991992

[CIT0011] Jonker, A., S. M. Hickey, S. J. Rowe, P. H. Janssen, G. H. Shackell, S. Elmes, W. E. Bain, J. Wing, G. J. Greer, B. Bryson, et al.. 2018. Genetic parameters of methane emissions determined using portable accumulation chambers in lambs and ewes grazing pasture and genetic correlations with emissions determined in respiration chambers. J. Anim. Sci. 96:3031–3042. doi: 10.1093/jas/sky187 29741677 PMC6095386

[CIT0012] Jonker, A., S. Hickey, J. C. McEwan and G. C. Waghorn. 2020. Portable accumulation chambers for enteric methane determination in sheep. In: Guideline for estimating methane emissions from individua ruminants using: GreenFeed, sniffers, hand-held laser detector and portable accumulation chambers. A Jonker, GC Waghorn,eds. New Zealand Agricultural Greenhouse Gas Research Centre: Palmerston North, New Zealand.

[CIT0013] Latif, M. G. A., and E. Owen. 1980. A note on the growth performance and carcass composition of Texel-and Suffolk-sired lambs in an intensive feeding system. Anim. Sci. 30:311–314. doi: 10.1017/s0003356100024120

[CIT0014] Intergovernmental Panel for Climate Change (IPCC). 2021.Climate Change 2021. In: The Physical Science Basis. Contribution of Working Group I to the Sixth Assessment Report of the Intergovernmental Panel on Climate Change. Masson-Delmotte, V., P. Zhai, A. Pirani, S. L. Connors, C. Péan, S. Berger, N. Caud, Y. Chen, L. Goldfarb, M. I. Gomis, M. Huang, K. Leitzell, E. Lonnoy, J. B. R. Matthews, T. K. Maycock, T. Waterfield, O. Yelekçi, R. Yu, and B. Zhou, eds. Cambridge University Press, Cambridge, United Kingdom and New York, NY, USA, 2391 pp.

[CIT0015] Mayes, R. W., C. S. Lamb, P. M. Colgrove. 1986. The use of dosed and herbage n-alkanes as markers for the determination of herbage intake. J. Agric. Sci. 107:161–170. doi: 10.1017/S0021859600066910

[CIT0016] McGovern, F. M., N. McHugh, S. Fitzmaurice, T. Pabiou, K. McDermott, E. Wall, N. Fetherstone. 2020. Phenotypic factors associated with lamb live weight and carcass composition measurements in an Irish multi-breed sheep population. Transl. Anim. Sci. 4:txaa206. doi: 10.1093/tas/txaa206 33409463 PMC7758996

[CIT0017] McGovern, F., B. Garry, P. Creighton, N. Galvin, D. Hennessy, E. Kennedy, N. McHugh, M. O’ Donovan, M. Beecher. 2021. Validating the n-alkane technique for determining herbage dry matter intake in sheep offered perennial ryegrass harvested at varying growth stages and seasons. Anim. Feed Sci. Technol. 279:115025. doi: 10.1016/j.anifeedsci.2021.115025

[CIT0018] McGovern, F.M., E. O’Connor, L. Farrell, E. Dunne, E. Wall, and N. McHugh. 2022. Measuring methane in sheep production systems–phenotypic factors affecting output. In Proc. 12th World Congress on Genetics Applied to Livestock Production. Technical and species orientated innovations in animal breeding, and contribution of genetics to solving societal challenges. 2944–2947.

[CIT0020] Moorby, J. M., H. R. Fleming, V. J. Theobald, and M. D. Fraser. 2015. Can live weight be used as a proxy for enteric methane emissions from pasture-fed sheep? Sci. Rep. 5:17915. doi: 10.1038/srep17915 26647754 PMC4673420

[CIT0021] Morgan, D. J., G. Stakelum, J. Dwyer. 1989. Modified neutral detergent cellulase digestibility procedure for use with the’Fibertec’system. Irish J. Agric. Res. 28:91–92.

[CIT0022] Morrison, S. J., J. McBride, A. W. Gordon, A. R. Wylie, T. Yan. 2017. Methane emissions from grazing holstein-friesian heifers at different ages estimated using the sulfur hexafluoride tracer technique. Engineering. 3:753–759. doi: 10.1016/J.ENG.2017.03.018

[CIT0023] Muir, S. K., N. Linden, A. Kennedy, M. I. Knight, B. Paganoni, G. Kearney, A. N. Thompson, and R. Behrendt. 2020. Correlations between feed intake, residual feed intake and methane emissions in Maternal Composite ewes at post weaning, hogget and adult ages. Small Ruminant Res. 192:106241. doi: 10.1016/j.smallrumres.2020.106241

[CIT0024] Muir, S. K., R. Behrendt, M. Moniruzzaman, G. Kearney, M. I. Knight, et al. 2021. Automated feeding of sheep. 2. Feeding behaviour influences the methane emissions of sheep offered restricted diets. Anim. Prod. Sci. 62:55–66. doi: 10.1071/AN20634

[CIT0025] O’Connor, E., N. McHugh, T. M. Boland, E. Dunne, and F. M. McGovern. 2021a. Investigation of intra-day variability of gaseous measurements in sheep using portable accumulation chambers. J. Anim. Sci. 99:skab132. doi: 10.1093/jas/skab132 34417802 PMC8379718

[CIT0026] O’Connor, E., F. M. McGovern, D. T. Byrne, T. M. Boland, E. Dunne, N. McHugh. 2021b. Repeatability of gaseous measurements across consecutive days in sheep using portable accumulation chambers. J. Anim. Sci. 99:skab288. doi: 10.1093/jas/skab288 34637520 PMC8782229

[CIT0027] O’Connor, E., F. M. McGovern, E. Dunne, S. J. Morrison, T. M. Boland, and N. McHugh. 2022. The impact of sire on variation in methane production and dry matter intake in sheep. In Proc. 12th World Congress on Genetics Applied to Livestock Production. Technical and species orientated innovations in animal breeding, and contribution of genetics to solving societal challenges. 2905–2908.

[CIT0028] O’Connor, E., F. M. McGovern, D. P. Berry, E. Dunne, J. C. McEwan, S. J. Rowe, et al. 2024. Comparison of greenhouse gas emissions from sheep measured using both respiration and portable accumulation chambers. Animal. 18:101140. doi: 10.1016/j.animal.2024.101140 38626708

[CIT0029] O’Neill, B. F., M. H. Deighton, B. M. O’Loughlin, F. J. Mulligan, T. M. Boland, M. O’Donovan et al. 2011. Effects of a perennial ryegrass diet or total mixed ration diet offered to spring-calving Holstein-Friesian dairy cows on methane emissions, dry matter intake, and milk production. J. Dairy Sci. 94:1941–1951. doi: 10.3168/jds.2010-3361 21426985

[CIT0030] Oddy, V. H., A. J. Donaldson, M. Cameron, J. Bond, S. Dominik, and D. L. Robinson. 2018. Variation in methane production over time and physiological state in sheep. Anim. Prod. Sci. 59:441–448. doi: 10.1071/an17447

[CIT0031] Paganoni, B., G. Rose, C. Macleay, C. Jones, D. J. Brown et al. 2017. More feed efficient sheep produce less methane and carbon dioxide when eating high-quality pellets. J. Anim. Sci. 95:3839–3850. doi: 10.2527/jas2017.1499 28992015

[CIT0032] Pelchen, A., and K. J. Peters. 1998. Methane emissions from sheep. Small Ruminant Res. 27:137–150. doi: 10.1016/s0921-4488(97)00031-x

[CIT0033] Pickering, N. K., V. H. Oddy, J. Basarab, K. Cammack, B. Hayes, R. S. Hegarty, J. Lassen, J. C. McEwan, S. Miller, C. S. Pinares-Patiño, Y. De Haas. 2015. Animal board invited review: genetic possibilities to reduce enteric methane emissions from ruminants. Animal. 9:1431–1440. doi: 10.1017/S1751731115000968 26055577 PMC4574172

[CIT0034] Pinares-Patiño, C. S., P. D’Hour, J. P. Jouany, C. Martin. 2007. Effects of stocking rate on methane and carbon dioxide emissions from grazing cattle. Agric. Ecosyst. Environ. 121:30–46. doi: 10.1016/j.agee.2006.03.024

[CIT0035] Pinares-Patiño, C. S., K. R. Lassey, R. J. Martin, G. Molano, M. Fernandez et al. 2011a. Assessment of the sulphur hexafluoride (SF6) tracer technique using respiration chambers for estimation of methane emissions from sheep. Anim. Feed Sci. Technol. 166-167:201–209. doi: 10.1016/j.anifeedsci.2011.04.067

[CIT0036] Pinares-Patiño, C. S., J. C. McEwan, K. G. Dodds, E. A. Cárdenas, R. S. Hegarty, et al. 2011b. Repeatability of methane emissions from sheep. Anim. Feed Sci. Technol. 166-167:210–218. doi: 10.1016/j.anifeedsci.2011.04.068

[CIT0037] Reintke, J., K. Brügemann, T. Yin, P. Engel, H. Wagner et al. 2020. Assessment of methane emission traits in ewes using a laser methane detector: genetic parameters and impact on lamb weaning performance. Arch. Anim. Breed 63:113–123. doi: 10.5194/aab-63-113-2020 32363232 PMC7191252

[CIT0038] Renand, G., A. Vinet, V. Decruyenaere, D. Maupetit, and D. Dozias. 2019. Methane and carbon dioxide emission of beef heifers in relation with growth and feed efficiency. Animals. 9:1136. doi: 10.3390/ani9121136 31842507 PMC6940808

[CIT0039] Robinson, D. L., J. P. Goopy, R. S. Hegarty, and P. E. Vercoe. 2010. Repeatability, animal and sire variation in 1-hr methane emissions and relationships with rumen volatile fatty acid concentrations. In: Proc. 9th World Congr. Genet. Appl. Livest. Leipzig, Germany.

[CIT0040] Robinson, D. L., J. P. Goopy, R. S. Hegarty, and V. H. Oddy. 2015. Comparison of repeated measurements of methane production in sheep over 5 years and a range of measurement protocols. J. Anim. Sci. 93:4637–4650. doi: 10.2527/jas.2015-9092 26523556

[CIT0041] Russel, A. J. F., J. M. Doney, and R. G. Gunn. 1969. Subjective assessment of body fat in live sheep. J. Agric. Sci. 72:451–454. doi: 10.1017/s0021859600024874

[CIT0042] Ryan, C. V., T. Pabiou, D. C. Purfield, S. Conroy, S. F. Kirwan, J. J. Crowley, et al. 2022. Phenotypic relationship and repeatability of methane emissions and performance traits in beef cattle using a GreenFeed system. J. Anim. Sci. 100:skac349. doi: 10.1093/jas/skac349 36268991 PMC9733524

[CIT0043] Swainson, N., S. Muetzel, and H. Clark. 2016. Updated predictions of enteric methane emissions from sheep suitable for use in the New Zealand national greenhouse gas inventory. Anim. Prod. Sci. 58:973–979. doi: 10.1071/an15766

[CIT0044] Ulyatt, M. J., K. R. Lassey, I. D. Shelton, and C. F. Walker. 2005. Methane emission from sheep grazing four pastures in late summer in New Zealand. doi: 10.1080/00288233.2005.9513671

[CIT0045] Van Soest, P. J. 1963. Use of detergents in the analysis of fibrous feeds. II. A rapid method for the determination of fibre and lignin. J. AOAC Int. 46:829–835. doi: 10.1093/jaoac/46.5.829

[CIT0046] Weston, R. H. 1988. Factors limiting the intake of feed by sheep. 11. The effect of pregnancy and early lactation on the digestion of a medium-quality roughage. Aust. J. Agric. Res. 39:659–669. doi: 10.1071/ar9880659

[CIT0047] Wims, C. M., M. H. Deighton, E. Lewis, B. O’Loughlin, L. Delaby, T. M. Boland, M. O’Donovan. 2010. Effect of pregrazing herbage mass on methane production, dry matter intake, and milk production of grazing dairy cows during the mid-season period. J. Dairy Sci. 93:4976–4985. doi: 10.3168/jds.2010-3245 20855032

[CIT0048] Zetouni, L., M. Kargo, E. Norberg, and J. Lassen. 2018. Genetic correlations between methane production and fertility, health, and body type traits in Danish Holstein cows. J. Dairy Sci. 101:2273–2280. doi: 10.3168/jds.2017-13402 29331458

